# On the Condition of Lunacy in England

**Published:** 1848-01-01

**Authors:** 


### ART. III.—*Report of the Commissioners in Lunacy to the Lord Chancellor.* 1847. Shaw and Sons.

THERE are few subjects that can engage the attention of the friend of humanity of more importance, and at the same time of more painful interest, than the condition of the insane. The feelings of every one who is in the enjoyment of that greatest of earth's blessings, the "mens sana in corpore sano," are enlisted on behalf of the poor soul who, deprived of nature's light and guide, is degraded below his species, and reduced to the level of the beast which perishes. Man's boasted prerogative denied, the hapless lunatic wanders frail and helpless on the shores of life, and depends for very existence on the sympathy of those more fortunate. His fellow-creatures are to him beings of a superior order, and have the power to treat him as they will. He is as an infant in their hands. But too often has this power been abused; and instead of being ministering angels to the outcast, to win him back to light and reason by kindness and protection, the imbecile has been pursued and harassed by tormenting demons, and driven further into the abyss of gloom. Surely there is a great responsibility attached to those who have the care of the forlorn. This we are pleased to see is now felt. The improvements that have successively taken place and the efforts still making to befriend this class of the wretched, are the surest signs of an enlightened age. Those names will stand prominently and most honorably forward in the history of civilization, which have been united with this noble cause. Philanthropy never was more needed or employed to better purpose.

Mainly to the exertions of a few individuals, in the face of great obstacles, has the condition of the insane been forced upon the legislature. To their zeal and persevering efforts do we owe, undoubtedly, the present provisions in their favour. Honour be to them! The general apathy on this subject would be unaccountable, were it not considered that the question is not even now properly understood by the public. If the advocates of the insane have, after all, failed to create any permanent interest in the subject, we must suppose that it has never been regarded in the light of a great national evil, spreading through numerous families, and calling for every remedy that medical science can suggest or law can enforce. Its extent and importance have been greatly underrated. The whole subject is most distasteful to the public, and a cold shudder runs through the frame at the bare thought of the wretched lunatic. It has never been sufficiently taken up as a matter of general interest; and has been rarely alluded to in the daily prints, except when the income of a patient, his fitness for liberation, or the cause of his death, have been made the subject of special inquiry, in the shape of a legal or equitable question.

The present report of the commissioners furnishes us with ample materials on some points, but is necessarily defective in others. This is chiefly attributable to the limited and partial nature of their jurisdiction: some large institutions for the insane not being subjected to their visitations, and the precise definition of the term, "person of unsound mind," not being by any means generally agreed upon.

Lunatics, in England and Wales, are dispersed about in various places. Some dwell in private houses under the special care of a medical or other attendant; others in houses especially licensed for their reception; while the great mass reside in hospitals and workhouses, or county asylums solely devoted to their accommodation. In forming an estimate of the numbers of persons of unsound mind no very accurate data exist, as the returns do not include a very numerous class of *imbecile* persons, whose infirmity has been coexistent with life, or is the result of the decay of natural powers. These persons should properly be included in our estimate, as they are frequently on the verge of idiotcy, and being incapable of managing their affairs in an efficient manner, require the same protection, if not the same restraint, as ordinary lunatics. Exclusive of these, there appear to be more than 23,000 persons of unsound mind scattered over the country; and of these, nearly 5,000 belong to the higher and middle classes of society, and about 18,000 are paupers.

The estimate formed by the commissioners, for the 1st of January of the present year, is to this effect :—

| | Persons. |
|---|---|
| 1. In the County Asylums, Hospitals, and Licensed Houses, 3,574 private patients, and 9,652 paupers ; together . . . . . . . . . | 13,226 |
| 2. In Bethlem, and in the Naval and Military Hospitals . . . . | 606 |

To these must be added,

| | |
|---|---|
| 3. Paupers in Poor-law Unions and places under local acts . . . | 8,986 |
| 4. Paupers in Gilbert's Unions, and other places not in union . . . | 176 |
| 5. Also 307 of 542 single patients, found lunatic by inquisition, (235 being in Licensed Houses) . . . . . . . . . | 307 |
| 6. Other single Patients in Private Houses, under the charge of persons receiving profit . . . . . . . . . . . . . | 130 |
| 7. The excess of Pauper Patients in Workhouses, &c., estimated by the Visiting Commissioners as, at least, one-third over the number returned by Parish Officers . . . . . . . . . . | 3,053 |
| 8. Criminals in Gaols . . . . . . . . . . . . | 32 |
| | 26,516 |

When we add to this number the many thousands of persons of various conditions of life, engaged in the care and protection of the insane,—some as committees and visitors, others as proprietors of licensed houses, and the rest as judicial officers, superintendents, matrons, clerks, stewards, male and female attendants, and domestic servants,—we find that there are, at least, 30,000 persons interested in the question of insanity.

The expense of the maintenance of these lunatic patients is estimated at near a million of money, according to the following table :—

| | £ | s. | d. |
|---|---|---|---|
| 1. The cost of 9652 Paupers in Asylums, estimated at an average of eight shillings per week each . . . . . . . | £200,761 | 12 | 0 |
| 2. Ditto of 8986 Paupers in Union Workhouses, &c., and 173 in Parishes not in union, (together, 9159,) estimated at an average of three shillings per week each . . . . . | 71,440 | 4 | 0 |
| 3. Ditto of excess of 3053 Paupers on the number returned by Parish Officers . . . . . . . . . . . . | 23,813 | 8 | 0 |
| 4. Ditto of 3574 Private Patients in Asylums, &c., at an average of twenty shillings per week each, (deducting the cost of 235, part of the 542 found lunatic by inquisition) . . | 173,628 | 0 | 0 |
| 5. Income of 542 Private Patients found lunatic by inquisition . | 280,000 | 0 | 0 |
| 6. Cost of 606 Patients in Bethlem, and the Naval and Military Hospitals, estimated at ten shillings per week each . . | 15,756 | 0 | 0 |
| 7. Cost of 120 other single Private Patients, taken charge of in separate houses, at £100 per annum each . . . . . | 12,000 | 0 | 0 |
| 8. Thirty-two Criminals in Gaols, estimated at three shillings per week each . . . . . . . . . . . . | 249 | 12 | 0 |
| | £777,648 | 16 | 0 |

To this amount is added the expense of maintaining many families cast upon the parish by the parents' insanity, the expense of supporting many persons termed "imbecile," and the interest of the large sums invested in the public lunatic establishments, (some of which are paying interest on borrowed money,) which together will raise the above expenditure of 777,648*l.* 16*s.* to little less than 1,000,000*l.* annually.

Thus the extent and importance of the subject should not be underrated, as the question affects, in one way or other, some 30,000 persons, and a yearly administration of a million of money, independently of its bearing upon the general liberty and welfare of the subject.

Reckoning 12,397 for Ireland, and 3413 for the pauper lunatics of Scotland; add to these the private patients in each country, with the various medical officers, attendants, &c., we find that the total number of individuals, who in Great Britain are, directly or indirectly, involved in the subject of lunacy, is little short of fifty thousand persons.

Except among a few benevolent individuals, the condition of the insane has created apparently no interest or inquiry. It seems clear in fact, that for a long series of years a large proportion of the lunatic poor more especially, must have altogether escaped the observation both of the government and the public. Their numbers were not known with any degree of correctness, even in their own counties or parishes. It is only of late years that any but the most scanty means have existed for enabling persons to judge of their general condition.

A parliamentary committee in 1807 first investigated the state of pauper lunatics. Other select committees investigated the condition of madhouses in 1815 and 1816; upon which latter occasions evidence was taken at great length as to the condition of Bethlem, St. Luke's, Guy's, and the York hospitals, and of some private lunatic establishments receiving paupers. And in 1827, another select parliamentary committee inquired into the state of the "madhouses in the county of Middlesex." Yet so late as the year 1827, the pauper lunatics of England, except such as were in county asylums, were left without any effective enactment in their favour.

We can infer the condition of the insane poor when left to the care of their parish officers, or who otherwise were without any care or protection whatever except such as might be afforded by their own families, by the dreadful condition of those patients who were confined in hospitals or licensed houses, which had the benefit of some supervision, and which became the subject of parliamentary inquiry and public comment.

So little indeed was the question understood, even by the legislature, that the first act which notices pauper lunatics (17 Geo. II. c. 5) enables any two justices to cause them to be apprehended, and to be locked up in some secure place, "*and there chained;*" and if the pauper's settlement should prove to be in another parish, then he was to be forwarded thither, and then "*locked up and chained,*" by the justices of that district. In fact, until the year 1828, no one was appointed to visit any of them, except such as were in county asylums, or to see that any care was bestowed upon them, or that they were not oppressed (as, in fact, they often were) by the cruelty of the attendants,—to whose almost unrestrained authority they appear to have been subject. Some of them, indeed, passed under the review of certain members of the College of Physicians, who visited the metropolitan licensed houses, a few of which included some pauper lunatics; but these visitors had, in truth, no power to remedy any abuse that they might discover in the establishments which they were directed to inspect.

It appears, by the evidence taken before the select committees, that the act of parliament "for regulating madhouses," which in terms directed visitations to be made to lunatics, was utterly useless as regarded private patients; and that its provisions such as they were did not even apply to the lunatic poor, who were sent to asylums without any medical certificate, and indeed without any authority, except that of their parish officers. Any person, whatever his character might be, was entitled to have a licence to receive lunatics. The act (14 Geo. III. c. 49) directed certain commissioners to meet for the purpose of granting licences; which licences, in the words of the section, "they are hereby required to grant *to all persons who shall desire the same.*" The commissioners, therefore, naturally complained that they had no power to effect any good whatever by their visitations. They had no power to refuse a licence, nor to control the person possessing one in any respect; nor, unless he refused them admission into his establishment, to inflict any punishment upon him. It was enacted indeed, that if the commissioners should discover anything deserving of censure, they should report the same to the College of Physicians (who itself had no power to punish the offenders), and that such part of their report should be hung up in the College, to be "perused by whosoever should apply for that purpose." This was the only power which the legislature gave them to correct abuses in madhouses. The commissioners knew nothing of removals, nothing of deaths; they had no power as respected the patients, either to liberate them when recovered, to remove restraint, to regulate the supply of food, to compel proper medical attendance, or in fact to enforce compliance with any suggestion which they might make, however important it might be for the comfort or even safety of the lunatic.

To remedy some of these defects,—to place some security round the pauper, as well as around the private patient,—and to provide frequent and efficient visitation to all,—a bill was framed and brought into the House of Commons, by Mr. Robert Gordon, and passed (with some amendments) on the 15th July, 1828, as the act 9 Geo. IV., c. 41. Important powers were given by this act. Amongst many other valuable provisions, it empowered certain commissioners within the metropolitan district and the justices in sessions throughout the provinces, to license all houses receiving two or more lunatics, "if they should think fit;" thus indirectly authorizing them, for the first time, to refuse a licence. It directed the commissioners, and certain justices (in their respective jurisdictions), to visit every house four times a year; and it made the concealment of a patient a misdemeanour. It enacted, that no private patient should be received without two medical certificates and no pauper patient without one. That notices of every admission, removal, and death, should be sent to the clerks of the commissioners and visiting justices; by which means an account is now necessarily rendered of every patient. That there should be a medical practitioner resident in large asylums; and that every smaller one should be visited twice a week by a medical attendant, who should report thereon and on the health of the patients. And, finally, it empowered the commissioners and justices to liberate patients who, in their opinion, were detained in any house without sufficient cause.

Previously to this act, the commissioners had no means of ascertaining the number of pauper lunatics, and had no authority to require any account of them. "We ask the keeper how many patients there are. I enter the names as he gives them to me; *and if we come within a few of the number, we think ourselves very well off.*" (Dr. Powell's evidence, Minutes, 1815.) This act was subsequently amended in some respects, and was eventually remodelled, and reproduced as the Act 2 & 3 Will. IV., c. 107; which gave more extensive powers, and assigned additional duties to the commission.

Since the report made by the Metropolitan Commissioners in Lunacy to the Lord Chancellor in the year 1844, two Acts of Parliament have passed and are now in force: the one for regulating the care and treatment of lunatics generally; and the other for the provision and regulation of lunatic asylums for counties and boroughs, and the maintenance and care of pauper lunatics therein. It is under these two acts that the present powers of the commissioners are exercised, and their various duties are imposed. A brief summary of these duties may be thus stated :—

By the first act (8 & 9 Vict., c. 100), the commissioners are authorized to hold boards or meetings, for licensing houses receiving lunatics and for other purposes connected with the commission, whensoever necessary. To visit all the county and borough asylums, gaols (containing lunatics), hospitals, and licensed houses in England and Wales; to inspect every part thereof,—to see every patient therein,—and to make certain inquiries relative thereto; the visitations to be made to the asylums, gaols, and hospitals once a year,—to the licensed houses in the provinces, twice a year,—and to the licensed houses within the metropolitan district, four times in each year. To examine and make entries in the several books kept at every hospital and licensed house throughout the kingdom; showing the result of their inquiries, the condition of the establishment, and the treatment of the patients therein at the time of each visitation; and also to make reports to the board as occasion may require. To visit all the parish and union workhouses in England and Wales, and the patients therein; to make certain inquiries there, and to report to the Poor-law Commissioners. Lastly, to inquire into the property of lunatics and to report thereon to the Lord Chancellor, and to examine into the cases of single patients.

The principal duties imposed upon the commissioners under the second act (8 & 9 Vict., c. 126), appear to be as follows:—1st. To receive and take into consideration all proposals and agreements for uniting counties and boroughs, and for building and providing asylums, buildings, yards, and other accommodation for pauper lunatics, and all contracts and plans intended to be adopted; to make inquiries relative thereto, and to report thereon to one of Her Majesty's principal Secretaries of State; and to make the same inquiries and report relative to all contracts for the maintenance of paupers in licensed houses. 2nd. To receive and make yearly abstracts of the accounts of all moneys received and paid on account of all county and borough asylums, for the purpose of the same being submitted to both Houses of Parliament.

These are the chief duties of the commissioners under the two acts, but there are still many obligations of a very onerous and multifarious kind, which do not come under either of these heads. These consist in reviewing and considering the sufficiency of all orders, medical certificates, notices, statements, and other returns transmitted to them upon the occasion of the admission, discharge, death, removal, and escape of every lunatic; the nature and sufficiency of every medical and other register required to be kept by each of the above-mentioned acts; the correctness of all licences granted in the provinces, and the suggestions of all visiting justices; together with many other matters, having reference to the condition of the various asylums receiving lunatics, the conduct of the proprietors, medical officers, and servants in those establishments, and the general welfare of all lunatic patients. Of course, all these matters have involved the necessity of extensive correspondence, and the frequent entry into long and difficult investigations.

It may be as well to add, that the present commission consists of Lord Ashley; Lord Seymour; the Right Hon. Robert Vernon Smith; Robert Gordon, Esq.; Francis Barlow, Esq.; Dr. Thomas Turner; Dr. John Thomas Hume; Dr. James Prichard; Bryan Waller Proctor, Esq.; James William Mylne, Esq.; and William George Campbell, Esq. To this board, Lord Ashley has been elected permanent Chairman.

We do not propose to discuss at this moment the merits of these acts, although relatively they are very great, but shall proceed at once to point out the abuses previously existing, and the necessity there was of speedy and vigorous legislation.

The enormities existing in asylums, public as well as private, previously to the parliamentary investigations of 1815, 1816, and 1827, can scarcely be exaggerated. In the words of the Report, " they comprise almost every species of cruelty, insult, and neglect, to which helpless and friendless people can be exposed, when abandoned to the charge of ignorant, idle, and ferocious keepers, acting without conscience or control." The management of these helpless beings was thus perhaps a little more private, but scarcely less barbarous, than that of the Turks at the present day; who fetter the poor maniacs up like wild beasts in public dens, where the boys can pull the chains and make sport of their rage and frenzy; or of the Peruvians, one of whose principal amusements it is to visit the public asylums in Lima on holidays, to mimic and torment the wretched inmates.

It is doubtful whether the fact of its being the interest of the proprietors of establishments, appropriated entirely to the reception of private patients, to satisfy the friends as to the treatment, operated sufficiently to prevent great oppression and injustice. We know that people were often actuated by cupidity and revenge in consigning their relations to these institutions. Their care and treatment therein would therefore be frequently a matter of indifference, and they would be left to the tender mercies of those who had their charge. Serious abuses doubtless existed there, as great may be as those in the houses mainly devoted to paupers.

The minutes of evidence taken before the several select committees appointed by the House of Commons in the years 1815, 1816, and 1827, furnish us with ample details as to the condition of the large lunatic establishments previous to the year 1828. From this source we learn the following particulars respecting the *York asylum.* On a public inquiry being instituted, it was found "that there were concealed rooms in the hospital, unknown even to the governors of the asylum; and that patients slept in these rooms, which were saturated with filth, and totally unfit for the habitation of human beings. Thirteen female patients were crowded in a room twelve feet by seven feet ten inches only: the keepers had access to the female wards, and several female patients became pregnant. One patient (a clergyman) was kicked down stairs by a keeper, while his wife was insulted by the keepers with indecent language, in order to deter her from visiting him. Another male patient disappeared, and was never afterwards heard of; four patients were supposed to be burned to death, (the asylum having been '*found to be on fire*,' a few days after a general investigation of it was directed;) and there were several other patients 'of whom no account could be given.'"

At this time, the physician was "the sole physician, sole visitor, and sole committee!" The food was bad; the asylum was bad throughout; crowded, ill-ventilated, and most dirty and disorderly. One patient, who had been kept for a week naked in a dark room full of filth, could only obtain a shirt by promising a bribe of five shillings to the keeper who was placed over him; and in fact the patients appear to have been left altogether to the caprice of ignorant and brutal attendants.

When *Bethlem Hospital* was examined in 1816, "female as well as male patients were chained to the walls, covered only with a blanket formed into something like a gown. One man (Norris, whose case is well known) was kept confined in chains for *fourteen years,* without the smallest interval of liberty. Stout iron rings were riveted round his arms, body, and neck, the latter being made to slide upwards and downwards on a massive iron bar inserted in the wall. And he was placed under the care of a keeper who was almost constantly drunk, but who nevertheless retained his situation several years. Patients were liable to be chained, not merely for safe custody but as a punishment. It would appear from the evidence that little or no medicine, with the exception of a certain "powder," was administered to the patients, 122 in number, and that the medical attendant did not reside in the hospital but came once a day for an hour. The system of treatment consisted of bleeding, purging, and vomiting, in the spring months. A certain day was appointed on which the patients were bled, another when they were purged, another when they were vomited. They were bled in May, and again in June: the precise time depended on the weather. All this had been the practice for many years. The patients were, at one time, left for ten years to the care of a surgeon, who was "generally insane, and mostly drunk."

It would appear, from the evidence, that at *St. Luke's Hospital* in 1815, the dirty patients were without a change of clothes, inasmuch as it was the custom to keep them in bed one day when "their things were washed and put on again." At this time, the master stated that he was never in the habit of keeping violent patients in bed above four or five days at a time. When in May 1816 the hospital was inspected, it was found that the walls were excessively filthy, not having been whitewashed for five years; the day-rooms were crowded, ill ventilated, and highly offensive; there was not half the proper number of attendants; there was no classification, and no employment.

At ——— *House*, in May, 1815, the dirty and clean patients were intermixed. Twelve of the males slept two in a bed. The rooms were crowded, wet, filthy, unventilated, and very offensive; some of the dormitories were lighted and aired by apertures without glass; and the patients themselves appear to have been altogether neglected, and to have been without medical treatment, or any proper care. Some of the patients had been there fourteen years, to whom a single grain of medicine had never been administered for the cure of their insanity. One poor object was exceedingly dirty, much emaciated from an affection of the chest, and had a wooden bowl before him, with a few dirty potatoes in it, but was without drink, medicine, or an individual creature to give him the smallest assistance.

The horrors seem to increase as we proceed further. On inquiry into the condition of two other asylums, it was discovered, that in the month of December, "several of the paupers were chained to their bedsteads naked, and only covered with a hempen rug;" and that "some pauper men were chained upon their straw beds, with only a rug to cover them, and not in any way defended from the external cold." In 1816, it was stated in evidence before the committee, that the patients were subjected to brutal cruelties from the attendants; that they suffered very much from cold, (one patient having lost her toes from mortification proceeding from cold;) and that they were infested with vermin. In 1827, it was further stated in evidence, that dirty patients were chained to their cribs, and confined without intermission from Saturday night until Monday morning, in crowded, ill-ventilated places; that the object of this was to give some of the keepers a holiday on Sunday; that the patients lay in these cribs naked upon straw, with nothing but a blanket to cover them, although the window was merely an aperture without glass; that there were dirty patients insensible to the calls of nature, yet that none of them were washed, and a few only of them were "wiped," during this period; and that on the Monday morning, even in November, (and, as one witness stated, in frosty weather,) they were rubbed down with a mop dipped in cold water, like so many animals. It was further stated that there was no medical treatment for insanity; that there was no system of employment or classification; and that the patients were entirely at the mercy of their keepers. It appeared (amongst other things), that for 170 male pauper patients there was only one towel per week allowed and no soap; that there was no medical resident; and that the house, although it contained nearly 500 patients, was visited only twice or three times a week by an apothecary, who merely prescribed strong doses of purgative medicines occasionally for the patients.

Pass we now from this truthful but repulsive picture to that more agreeable one presented by the present condition of the same asylums. The *treatment* of insanity is doubtless much better understood now than it was formerly, but still much good has been effected by public attention having been directed to them. There can be no question but that the receptacles for lunatics in this country have undergone great though gradual improvement during the last few years. The dwellings for the insane are no longer the gloomy prisons in which they were formerly confined; cleanliness, warmth, and ventilation, are insisted on; better diet, clothing, and bedding, have been provided; personal restraint is diminished, and even when still employed its severity is greatly mitigated and its application strictly watched. The health and mental condition of the lunatic are more carefully considered, occupation and amusement are more generally afforded to him, and in all respects better treatment is secured, whilst an opportunity is periodically given him of representing any hardship to which he may have been subjected.

Allowing, as we have said, for the better system of medical treatment, it would scarcely be too much to affirm that the greater part of this evident improvement in the condition of these establishments is due to the special supervision to which they are subject. It is the duty of the commissioners to ascertain that the patient is duly confined; that he has medical aid, fit attendance, and proper comforts, during his confinement; that he is provided with employment and amusement; that his food is good, and his place of residence healthy, clean, well ventilated, and in good order; that he himself is not ill-treated, neglected, or improperly restrained; and, finally, that he is liberated when fit for liberation. Under a supervision of this kind, evils such as existed previously to the year 1815 must necessarily be extinguished. The commissioners accordingly, in their report, speak favourably on the whole of the present condition of both public and private asylums. In order to show what good may be effected in an asylum originally bad in almost every respect, by supervision carefully and regularly made, and where the medical attendant is skilful and willing to attend to useful suggestions, and the proprietor is liberal enough to carry them out at any reasonable expense,—the following case may be cited from the report. It is also a good specimen of a well regulated establishment for a large number of patients. The various improvements that have been gradually going on in this asylum since the last parliamentary inquiry are thus stated:—

"1. An active and able medical superintendence has been established, under which every suggestion of the commissioners for improving the asylum and benefiting the patients, has been at all times readily attended to.

2. The excessive use of mechanical restraint has been abolished, and restraint itself reduced to the minimum degree consistent with the safety of the patients. In place of there being seventy patients in irons, the number now subjected to restraint is exceedingly small, there being sometimes only one or two, and occasionally no patient whatever, under any mechanical coercion.

3. From having no baths whatever, there are now warm and cold baths and shower baths; and from cleanliness being utterly neglected it is now studied carefully, with a view to the health as well as comfort of the patients. There are conveniences (within doors) for washing attached to every yard; as much soap and towelling as the attendants require is distributed; baths are used weekly, and every patient is washed regularly every day.

4. The day-rooms and dormitories are now clean and of good size, (the latter amply supplied with good bedding,) and there are large infirmaries, warm and well ventilated, for the sick and infirm. The whole of the premises have been drained, every yard having a barrel drain of two feet diameter with a constant run of water, and every water-closet communicating with the drain.

5. From there being no library, it will be seen, first,—That books are purchased; that these are placed under the care of a patient, and that all the patients (pauper as well as private,) have access to them. In 1835, it appears, that a library of 500 volumes had been collected; in 1837 it consisted of 600 volumes; in 1844, of 1200 volumes; in 1845, of 1500 volumes; and at present, we understand that it consists of 2000 volumes of books, which are accessible to all classes of patients and are much used.

6. From possessing no amusements, the patients have now cards, skittles, bagatelle tables, backgammon-boards, &c.; a billiard table has also been provided for them, and a billiard-room has been erected for their use. And from there having been little or no employment, the patients of both sexes are provided with materials for occupation;—some are placed in the garden; others in the laundry, in shops, or in needle-rooms. A loom is erected; tailors', shoemakers', carpenters', and papier-maché shops have been established. The patients are encouraged by small gratuities to employ themselves; and a considerable proportion, fluctuating from time to time, but amounting sometimes to 150 or more of each sex (or nearly three-fourths of the patients), are employed in the asylum. And it is stated that this system of employment diminishes the necessity for restraint.

7. From there having been three nurses only for 154 patients, being at the rate only of one for fifty patients, there is now, reckoning the entire establishment, one attendant for every fifteen patients.

8. The dietary is good and ample, and has never within our recollection been complained of. Good joints of meat, and good vegetables only are purchased; and the attendants are ordered (as one of the "General Rules" of the asylum,) to supply more food whenever a patient asks for it, except only in cases of morbid appetite.

9. The entries will show that, at the suggestion of the commissioners, rooms have repeatedly been disused, new rooms erected, and a variety of improvements made; and that for promoting good classification the whole arrangement of the houses has been altered. The whole of one house has been taken down and rebuilt. The rooms are now spacious and cheerful, and kept clean, warm, well ventilated, and comfortable. Since the act passed in 1828, between 22,000*l.* and 23,000*l.* have been expended in rebuilding and improving the establishment."

When it is added, that this account relates to certain houses, on account of the wretched management of which the metropolitan commissioners contemplated discontinuing the licence (the horrible barbarities committed therein were previously alluded to), we can form an idea of the change effected. In the other asylums also the chief abuses have been corrected, and the course of reform is even still in progress. In the York asylum, for instance, the governors at every quarterly court appoint five members of their body, who inspect the condition of the house and of the patients once every month; and they also appoint a committee of eight visitors, four gentlemen and four ladies. The special business of this committee is to make their rounds systematically through every part of the house; to see and converse with the patients, to listen to their complaints, and to inquire into all things connected with the management or general arrangement of the hospital, which may seem to require observation or correction. They visit, not at any stated periods, but at all hours and seasons, sometimes together and sometimes singly; and the result of their observations is recorded in a report book which is laid before the governors at the next quarterly court. This asylum is now in a most creditable condition. At St. Luke's, and elsewhere, equal benefit has been effected. A suitable number of medical and other attendants has been provided. Various day-rooms, dormitories, infirmaries, and domestic offices, have been erected; restraint has been in a great measure abolished and the diet rendered plentiful and wholesome. As to Bethlehem Hospital also, which is specially excepted from the visitations of the commissioners, it is said that it now exhibits none of the barbarities which were formerly practised within its walls. The patients are now placed under a regular medical staff, and everything is well ordered.

These facts tend to show how advantageous, and indeed how necessary, is the frequent visitation of all asylums. A careful and rigid scrutiny has been found to contribute more than anything else to ensure comfort and cleanliness in lunatic establishments, and good treatment to the insane. The aggregate amount of benefit derived by the patients, from the amendment of the defects pointed out by the commissioners has undoubtedly been very great.

Nor has less good been effected in private asylums. The power of withholding the licence from these establishments must operate and has operated, as a powerful incentive to the proprietors to attend to the suggestions of the commissioners. In some cases the licence has been withdrawn, and the houses have consequently been discontinued. In others, threats have been held out that such a measure would be resorted to, and the hope of renewing it has always operated as a powerful moral agent. The views of the commissioners on the subject of their visits to these establishments are thus alluded to in their report:—

"It is indispensable that powers of supervision should exist in every case; that they should be vested in persons totally unconnected with the establishment; and that the visitations should not be limited in point of number, and should be uncertain in point of time; for it is most important to the patients, that every proprietor and superintendent should always be kept in expectation of a visit, and should thus be compelled to maintain his establishment and its inmates in such a state of cleanliness and comfort as to exempt him from the probability of censure. We are satisfied from our experience, that if the power of visitation were withdrawn, all or most of the abuses that the parliamentary investigations of 1815, 1816, and 1827, brought to light would speedily revive, and that the condition of the lunatic would be again rendered as miserable as heretofore."

In the whole of this we cordially agree.

In consequence of this careful supervision, the commissioners are able to report that, upon the occasion of their recent visits to the various asylums, they have found that, with some exceptions (which they are exerting themselves to rectify), the patients are humanely and sometimes very judiciously treated. As they are in the habit of making frequent inquiries of the patients themselves, of listening to their complaints, and of redressing them, if in their opinion they are well founded: they think they may assert, from the infrequency of their occurrence and from the result of their investigations, that there is no reason to apprehend that the lunatic patient is now often subjected to cruelty or ill treatment. Cases of neglect may undoubtedly sometimes occur, though seldom they believe attended with serious consequences. Cases manifesting a high degree of dereliction of duty have rarely come to their knowledge, and in these instances prosecutions against the offenders have been instantly and vigorously instituted.

They very properly remark:—

"As, however, lunatic patients are placed very much at the mercy of their attendants, it is most desirable to secure, as far as possible, persons of humane and respectable character as attendants on the insane, in every asylum throughout the kingdom."

In order to promote this laudable object, they have established a central register at their office, and requested the superintendent or proprietor of every lunatic establishment to forward to them the names of all male and female attendants employed by him, and to transmit notice whenever any attendant shall be engaged or dismissed, or shall quit the asylum, together with the cause of every dismissal. Such a register will undoubtedly be the means of very useful reference, and by extending the opportunities of ascertaining and verifying every attendant's character, operate most materially to ensure good conduct on their parts, and may eventually induce persons of superior qualifications to become members of this very useful class.

After thus stating in general terms the condition of the insane, we come now to investigate special subjects, and to see what improvement has taken place in points on which the public may be supposed to take an especial interest. Foremost among these is the article of *restraint*, the diminution or total abolition of which forms one of the most striking features of modern improvement.

It would appear that, previous to the parliamentary investigation, instances occurred where as many as seventy patients were under restraint at one time at a single establishment. In one asylum thirteen out of fourteen were in fetters or handcuffs. So late as 1830, there were *twenty* patients at an asylum in Sussex, of whom *eleven* were under restraint by day and *six* by night. At another period of the same year, of *eighteen* lunatics *nine* were confined; and in 1831 *ten* patients were under restraint out of *twenty-two*. So that it would appear, that even after the act which passed in 1828 came into operation and the asylum was regularly visited, mechanical restraint was found to prevail to the extent of fifty per cent. in that establishment. It is true that, at this period, the relative number was considerably less in some other provincial asylums, but still the proportion was in all enormous.

From an examination of the tables published in the report, we find that the instances of mechanical restraint at the present day are very few in number in the public asylum. Even in licensed houses the practice of coercion is an exception to the general rule of treatment; and the modes of restraint now adopted are such as to pain and irritate the patient as little as possible. The massive bars, rings, and chains of iron, formerly resorted to, are no longer seen. Long continued coercion is not permitted. Coercion itself is scarcely ever allowed except with the sanction of the medical officer, who is himself compelled by the act of parliament to record every week in a journal framed for the purpose, the name of every patient under restraint and in seclusion, and the means by which such restraint is effected. Even the *duration* of restraint must in some instances be specified. The journal is open to the inspection of every commissioner or justice visiting the house, which if in the provinces ought to be six times a year; and which if in the metropolitan district is generally visited at least that number of times if it contain paupers, or if there be reason to suspect that undue restraint is had recourse to;—thus the safeguards against lunatic patients being subjected to harsh or unnecessary restraint, from the cruelty, idleness, or caprice of their attendants, have been multiplied, and the chances of abuse reduced to a small amount. In those institutions where the greatest abuses in this respect prevailed, wonderful amelioration has been effected. The asylum in Sussex no longer exhibits instances of such excessive coercion; and where, as above mentioned, seventy persons were under restraint at one time, there are now to be seen but one or two only.

The *body-clothing* of the pauper patients is, according to the report of the commissioners, sufficient for warmth, and on the whole tolerably good; except in the cases of lunatics addicted to destroy their clothes (a frequent mark of insanity), and of dirty patients, in whom it is always extremely difficult with the utmost care to preserve any appearance of neatness.

The commissioners are also extremely particular in the inspection of the *bed-clothing*, which was formerly extremely scanty and insufficient in cold weather. It is still observed to be insufficient. In order to ascertain the quantity allowed to the patients as well as its condition in point of cleanliness, it is the custom of the commissioners on visiting the various asylums, especially in severe or damp weather, to cause the bed-clothes of many of the beds to be thrown open; and in all cases where a deficiency has been noticed, they have been most peremptory in their directions that additional covering should be immediately supplied.

By the eighty-second section of the act 8 and 9 Vict. c. 100, the commissioners are empowered to regulate the *dietary* of the pauper patients. This is a most important matter and subject of anxious inquiry, as not only the bodily health and comfort of the lunatics are concerned, but frequently the cure of his mental affliction is dependent on a judicious and plentiful supply of food. On this subject, we believe, the whole of the profession is agreed.

Much time and attention it appears has been devoted to this point, and deep consideration has been paid to the usual habits of the poorer classes, as respects the ordinary variety and abundance of their food, and also the chance of some articles failing or becoming less abundant. In order to avail themselves of their power if required, the commissioners have applied for copies of all the several dietaries now in use in establishments receiving pauper lunatics. On two occasions only have they thought it necessary to interfere, but have generally found any suggestions they may have offered promptly attended to. They therefore express themselves generally satisfied.

In truth, considerable caution is required in interfering in such a matter, as much must be left to the judgment and discretion of the superintendent. No general house-diet can be applicable to all the cases. Those who are actively employed, or who are old or in infirm health, require better and different food from those who are either unwilling to work or are incapable of any occupation. In the best regulated establishments accordingly, we find large supplies of extra nourishment given to those who require it. In one institution there is consumed by the patients, over and above the ordinary dietary—

"Seventy-two gallons of ale, and seventy-two gallons of porter every week. Those who work are allowed, in addition, tobacco and snuff and small gratuities in money. The aged and infirm also have extra allowances and more delicate food, including lunch daily, puddings, sago, arrow-root, &c., frequently; and, upon an average, 300 eggs, eighty-five fish dinners, and 210 ounces of wine and brandy weekly. Tea and coffee are also allowed, both to those who work and to all those who are old or infirm."

All this is as it should be. In fact, there appears to be no dearth or stinting of the dietary on the part of the conductors of the asylums, except such as is necessarily imposed by the present vile system, on the part of the parish officers, of farming out the unfortunate paupers. The amount of charge made at some particular house determines the question but too frequently, whether the lunatic shall be sent there or to some other establishment, without reference to the comparative comfort and good treatment which the patient may expect at the different asylums. The sums expended in providing a comfortable house, airing grounds, good food, and regular medical aid, (without which advantages the patient has small chance of cure,) and also a sufficient staff of respectable attendants and domestic servants, must always be very considerable, and entitles the proprietor to adequate remuneration. The highest rate paid by the parishes for the maintenance of their pauper poor is but *eight shillings a week;* and this to include board, lodging, clothing, medicine, and attendance. Little enough one would think; yet it appears that this sum is often considered extravagant. In one instance, the parish of St. Denis, Walmgate in York, required that it should be reduced to seven shillings; and on the proprietor's refusal, their pauper was removed to a cheaper place. In another case, the parish of St. Cuthbert, in York, objected to pay this sum: and finally the mother of the patient (herself very poor) was obliged to contribute one shilling weekly, in order that her daughter might have the comforts of a respectable asylum. Two paupers belonging to the Porklington Union were removed some little time back from the Dunnington-house Asylum, because the proprietor declined to keep them and to provide them with all necessaries for *six shillings a week.* The commissioners justly observe that, considering the present price of provisions, this is too bad; and have directed the attention of the visiting justices to the fact, requesting them to exert their influence with the guardians of the union, to induce them to make a more liberal payment. We may add our own opinion, that such endeavours to sail so close to the wind are after all but a false economy, as the lunatics have not a fair chance of recovery, and thus are likely to continue dependent much longer on the funds of the parish.

The *medical treatment* of the patients is now most judicious, and fully up to the present advanced state of science. There is ample evidence to prove, that the number of recoveries from insanity is in proportion to the degree in which these curative resources have been employed. We are rather surprised however to find that so few, comparatively, are under treatment at the asylums; but suppose this is to be accounted for by the large proportion of chronic cases dependent on organic change, which hold out no prospect of cure.

Lastly, let us inquire to what *extent* lunatics are now *benefited* by their admission into the asylums provided for their use. And this seems to depend—for no doubt can exist as to the benefit of medical treatment in many cases—on the removal of the patients to such establishments as soon as possible after the commencement of their disease. The question relates mainly to pauper lunatics, whose early or late admission into proper asylums determines for the most part the number of cures and the amount of mortality that occur in lunatic asylums. The number of recent curable cases admitted into asylums is still comparatively small. The majority consists either of chronic cases, of persons in a confirmed and hopeless state of mental alienation, or of others who arrive in broken down or feeble health, reduced by poverty and neglect or exhausted by disease; and thus the amount of cures becomes necessarily low, whilst the rate of mortality is alarmingly increased. From abundant evidence to this effect, it would seem that the amount of mortality from time to time occurring in lunatic asylums, depends most materially on the condition of the patients at the time of their admittance, (many of them, often the greatest number, being received in a dying or hopeless state;) and that the number of cures depends, not as might be supposed entirely or even principally upon the skill of the medical attendant, or the comforts afforded to the patients while under treatment, but on the conduct of the various officers of unions and parishes, whose duty it is to provide promptly and efficiently for the welfare of the lunatics;—and whose want of prudence and humanity has without doubt tended in numberless cases to render the disease permanent, and has thereby increased the burdens of their parishes more than all the accidental causes to which insanity is referable. The following extract from the evidence of a medical superintendent of a county asylum, contains very sensible remarks on the usual neglect of the symptoms of what has been properly styled "the incubation of insanity:"—

"From the duration of the disorder previously to admission, it may be inferred that either the premonitory stage was not understood or was altogether disregarded. Perhaps in some cases from the insidious and obscure development of the symptoms, the moral and intellectual changes noticed were not considered precursors of insanity. The accession of the malady has been erroneously dated from some sudden outbreak of violence, of incessant restlessness, or of extreme incoherence; whereas these symptoms, instead of merely announcing the commencement of some grave malady, may but too frequently be regarded as the premonitory symptoms of a fatal issue, the termination of a long continued series of changes originating in disease of the brain."

We fully concur with the commissioners that the office of visitor to a public or private lunatic asylum is often an onerous and disagreeable task, and that those who, from motives of benevolence, perform such a duty, deserve the thanks of the public. The necessity of such visitations is apparent, from the great good already effected by them. That they have been regularly made, both by commissioners and magistrates, we have every reason to believe. These gentlemen are fully aware of the great power in their hands, and we trust to their good sense to use it judiciously. The discretion they have used in granting, withholding, and revoking licences has tended to ensure well-situated, comfortable, and spacious premises, and humane and intelligent superintendents and servants to the establishments. Their exertions have been successfully employed in promoting the comfort and forwarding the cure of the unfortunate lunatic, and effecting his liberation when fit for discharge. On this latter point perhaps there is less need of supervision in respect of county asylums, where there is no inducement to detain a lunatic beyond the time requisite for his cure, where committees regularly inspect the premises, and where matters connected with the asylum are frequent matters of discussion in open session. But in regard to other establishments, and more particularly to licensed houses, the necessity of frequent visitations is obvious.

The judgment of the commissioners is nowhere more apparent than in the pains they take that the proprietor of a proposed establishment should not only be of good character and humane disposition, but also that he should possess sufficient pecuniary resources. Poverty, they are aware, leads often to the blackest crimes. Still, out of the nettle the flower safety may be plucked. A good and worthy man from the best and purest motives may undertake the management of the insane: as he then has the power—the great, the philanthropic, the noble power —of aiding the cause of humanity, by sympathizing with the weak, relieving the distressed, and, like his better angel, ministering to the restoration of his wretched but sorely afflicted brother.

